# Spatial trends and projections of chronic malnutrition among children under 5 years of age in Ethiopia from 2011 to 2019: a geographically weighted regression analysis

**DOI:** 10.1186/s41043-022-00309-7

**Published:** 2022-07-05

**Authors:** Binyam Tariku Seboka, Samuel Hailegebreal, Tizalegn Tesfaye Mamo, Delelegn Emwodew Yehualashet, Girma Gilano, Robel Hussen Kabthymer, Helen Ali Ewune, Reta Kassa, Mary Abera Debisa, Mulugeta Namaro Yawo, Habtamu Endashaw, Abel Desalegn Demeke, Getanew Aschalew Tesfa

**Affiliations:** 1 School of Public Health, College of Health Science and Medicine, Dilla University, P.O. Box 419, Dilla, Ethiopia; 2 Department of Health Informatics, Arbaminch University, Arbaminch, Ethiopia; 3 Department of Public Management and Policy, Dilla University, Dilla, Ethiopia; 4 Department of Nursing, Dilla University, Dilla, Ethiopia

**Keywords:** Chronic malnutrition, Stunting, Under-five children, Spatial, Geospatial, Geographically weighted regression (GWR), Multiscale geographically weighted regression (MGWR), Ethiopia

## Abstract

**Introduction:**

Undernutrition is a serious global health issue, and stunting is a key indicator of children's nutritional status which results from long-term deprivation of basic needs. Ethiopia, the largest and most populous country in Sub-Saharan Africa, has the greatest rate of stunting among children under the age of five, yet the problem is unevenly distributed across the country. Thus, we investigate spatial heterogeneity and explore spatial projection of stunting among under-five children. Further, spatial predictors of stunting were assessed using geospatial regression models.

**Methods:**

The Ethiopia Demographic and Health Surveys (EDHS) data from 2011, 2016, and 2019 were examined using a geostatistical technique that took into account spatial autocorrelation. Ordinary kriging was used to interpolate stunting data, and Kulldorff spatial scan statistics were used to identify spatial clusters with high and low stunting prevalence. In spatial regression modeling, the ordinary least square (OLS) model was employed to investigate spatial predictors of stunting and to examine local spatial variations geographically weighted regression (GWR) and multiscale geographically weighted regression (MGWR) models were employed.

**Results:**

Overall, stunting prevalence was decreased from 44.42% [95%, CI: 0.425–0.444] in 2011 to 36.77% [95%, CI: 0.349–0.375] in 2019. Across three waves of EDHS, clusters with a high prevalence of stunting in children under 5 years were consistently observed in northern Ethiopia stretching in Tigray, Amhara, Afar, and Benishangul-Gumuz. Another area of very high stunting incidence was observed in the Southern parts of Ethiopia and the Somali region of Ethiopia. Our spatial regression analysis revealed that the observed geographical variation of under-five stunting significantly correlated with poor sanitation, poor wealth index, inadequate diet, residency, and mothers' education.

**Conclusions:**

In Ethiopia, substantial progress has been made in decreasing stunting among children under the age of 5 years; although disparities varied in some areas and districts between surveys, the pattern generally remained constant over time. These findings suggest a need for region and district-specific policies where priority should be given to children in areas where most likely to exhibit high-risk stunting.

**Supplementary Information:**

The online version contains supplementary material available at 10.1186/s41043-022-00309-7.

## Introduction

Undernutrition is a severe global health problem, but it is particularly prevalent among children under the age of five in Sub-Saharan Africa (SSA) [1–4]. Height-for-age or more commonly, stunting, is a measure of linear growth, and children whose height-for-age is fewer than two standard deviations below the reference population's median (− 2 SD) are deemed short for their age or stunted [5, 6]. It is a key indicator of children's nutritional status which results from long-term deprivation of basic needs. Moreover, the effect of these chronic malnutrition measures is linked with impairments that continue throughout the life of a child [7–9], and also, it poses a significant threat to the future growth of a nation and the financial and general burden of healthcare. Eventually improving nutrition contributes substantially to attain the Sustainable Development Goals (SDG) aim of improving nutrition, and also to ensuring healthy lives, and promoting well-being by 2030 [10, 11].

Globally, the burden of under-five stunting has declined steadily from 203.6 million in 2000 to 149.2 million in 2020 [2–4, 8]; however, these figures show that chronic malnutrition is still prevalent on a huge scale, and faster progress is needed to reach the 2030 target of ending all forms of malnutrition. Ethiopia, the largest and most populous country in Sub-Saharan Africa, has the greatest rate of stunting among children under the age of five [12]. The prevalence of stunting in Ethiopian children is quite high, with about 57% of children under the age of 5 suffering from stunting in 2000 [13]. Through years of intervention efforts by the government and other stakeholders had shown remarkable progress, yet remained as high as 37% of children under 5 are short for their age or stunted (below − 2 SD) in Ethiopia, 2019 [14]. This staggering estimate of childhood stunting contravenes the goal of SDGs, and its reduction contributes to the country's progress toward achieving SDGs targets [10, 11]. However, despite having the highest burden of childhood stunting, it affects the country in a heterogeneous manner. For example in 2019, the disparities range from just 14% in Addis Ababa to 49% in Tigray [14]. Overall, it is evident from the existing evidence that there is a huge gap to achieve the global targets and needs more investigations by policymakers and decision-makers.

Mapping the trend and variation of chronic malnutrition among children under the age of five can aid in strengthening programs for allocating limited resources to districts with high healthcare needs [15–17]. To reach the goal of ending all forms of malnutrition as a public health threat by 2030 [5, 10, 11], it is critical to understand the epidemiology of under-five stunting and disparities in a region-specific manner and appropriately target high-risk populations in Ethiopia. In SSA, various studies indicated the spatial variation of stunting among under-five children [18–23]. Similarly, studies conducted in Ethiopia have also shown the spatial variation of all forms of malnutrition [24–31]. However, spatial studies on stunting among children under the age of 5 years are limited to showing the overall trend and lack modeling of spatial relationships between the identified clusters of stunting and its predictors. The present literature indicates that examining the spatial distribution of childhood chronic malnutrition in Ethiopia at the district level is necessary. The spatial predictors of stunting include factors from the previous literature [4, 17, 18, 26, 32], particularly UNICEF's general conceptual framework for causes of malnutrition [33] and the Lancet Nutrition Series [34].

Based on the Ethiopian Demographic and Health Survey (EDHS) conducted in 2011, 2016, and 2019, the study's main goal was to look at how stunting has changed over time among children under the age of 5 years in different districts and regions of Ethiopia. It also aimed to explore the spatial distribution of stunting across areas and zones, as well as to identify spatial clusters with high and low stunting prevalence. Furthermore, spatial regression modeling was employed to reveal spatial predictors of observed hotspots in the under-five stunting. Further, to assess the local relationships of under-five stunting predictors in Ethiopia, we have employed geographically weighted regression (GWR) and multiscale geographically weighted regression (MGWR) approaches.

## Methods

### Study design and setting

Data for this study were extracted from three waves of the Ethiopian Demographic and Health Surveys (EDHSs), which were conducted in 2011 [35], 2016 [36], and 2019 [14]. Ethiopia is the second-most populous country in Africa, with 110,613,986 people [37]. It is located in the horn of Africa. These cross-sectional surveys were conducted in Ethiopia's Afar, Amhara, Benishangul-Gumuz, Gambella, Harari, Oromia, Somali, Southern Nations, Nationalities, and People's Region (SNNP), and Tigray as well as two city administrative divisions (Addis Ababa and Dire Dawa).

All EDHS surveys used a stratified two-stage cluster sampling approach, and the adopted multistage sampling procedure involves the selection of clusters in the first stage and then the selection of households in the second stage for all EDHS surveys. Clusters were also divided into categories based on where they lived (rural vs. urban) and whose districts they came from (Woreda). Each Woreda is subdivided into Kebeles, which are subdivided further into census enumeration zones (EAs). Selected enumeration areas additionally included spatial information or location data (latitude and longitude coordinates). The study's design and setting are described in greater detail elsewhere [14, 35, 36].

### Source and study population

The source population consisted of all children under the age of 5, in Ethiopia. The study population consisted of children under five years of age who were selected for anthropometry measurements. The data from a total of 26,308 children aged below five years were analyzed for this study covering different surveys conducted in each of the following years: 2011 (*n* = 10,883), 2016 (*n* = 10,376), and 2019 (*n* = 5278).

### Tools and measurement

#### Variables of the study

The study outcome, stunting, was defined as a height-for-age that was less than 2 SD of the median height-for-age, according to WHO international growth criteria (i.e., HAZ <  − 2) [1, 5]. We employed sampling weights and a stratified sample design to construct estimates, confidence intervals, and standard errors with STATA to retain the representativeness of sampled data.

The year of the survey, as well as the region and zone in which the child lived, was included as independent factors in this study. Furthermore, spatial regression factors that have been associated with under-five stunting and the key predictors of stunting in Ethiopia were selected from the previous literature, particularly UNICEF's general conceptual framework for causes of malnutrition [33] and the Lancet Nutrition Series [34]. Factors such as the mother’s education, age of marriage (at least 18 years old), sanitation, wealth index, and family size were considered from socio-demographic factors. In addition, markers for maternal nutrition and child feeding include early commencement of nursing, exclusive breastfeeding, timely introduction of complementary foods, and an adequate diet. Factors were related to nutrition-specific interventions such as antenatal care (ANC) during the first trimester, adequate ANC (at least four ANC visits), and iron and folic acid (IFA) consumption. Full immunization, vitamin A supplementation, and oral rehydration solution during diarrhea were all indicators related to children’s health care.

### Data management and analysis

#### Statistical analysis

This study used multiple spatial statistical models to analyze the geographical variations and trends of stunting among Ethiopian children under the age of five. Furthermore, we have employed global ordinary least squares (OLS) to assess the global relationships of under-five stunting predictors. Further, at the local scale, we employed two modeling approaches: geographically weighted regression (GWR) and multiscale geographically weighted regression (MGWR). In this study, STATA version 14.2 was utilized for descriptive analysis. ArcGIS version 10.8 software was used for visualization, exploration, and spatial modeling, and maps were created for visual depiction of stunting among children under the age of 5 years in Ethiopia at the regional and district levels. Also, MGWR version 2.1 software was utilized for local spatial regression analysis. The CSA (Central Statistical Agency) database provided the Ethiopian district delineation shape file.

#### Spatial autocorrelation analysis

Spatial autocorrelation analyses were conducted to detect the geographical distribution of stunting among children under the age of 5 years in Ethiopia for each survey from 2011 to 2019. The spatial autocorrelation tool determines the spatial cluster or dispersion of under-five stunting or otherwise of the feature in the study setting by using the Global Moran’s I Index. The value of Moran’s I range between minus − 1 and 1, with a score of zero indicating no clustering and a positive score indicating a spatial concentration of similar values. The null hypothesis is rejected when Moran's I is statistically significant (*p* 0.05), indicating the presence of spatial autocorrelation [38].

#### Hot spot analysis (Getis–Ord Gi* statistic)

The Getis–Ord Gi* statistics were used to identify the location and variation in the degree of clustering for each area. For each location in the data set, Getis and Ord's distance-based Gi and Gi* statistics are computed as the ratio of the sum of the values in neighboring locations to the sum of all the values in the study region. The *p* value is computed for the significance of under-five stunting clustering, and the *Z*-score is utilized to test its statistical significance. The *p* value associated with a 95% confidence level is 0.05. If the *z*-score is between 1.96 and + 1.96, and the *p* value is more than 0.05, then the null hypothesis cannot be rejected; the pattern displayed is most likely the consequence of random spatial processes. If the *z*-score is outside the range, the observed spatial pattern is likely too unique to be due to chance, and the *p* value will be tiny to reflect this. In this scenario, the null hypothesis may be rejected and the investigation can focus on determining what is driving the statistically significant spatial pattern in the data. A high Gi* statistical output suggests a “hotspot,” while a low Gi* indicates a “cold spot.” [39].

#### Spatial interpolation

The spatial interpolation technique was used to predict the unsampled from observed spatial patterns of stunting in children under the age of five. In this study, the kriging spatial interpolation method was utilized to make predictions and smooth surfaces of the geographical distribution of under-five stunting [40].

### Modeling spatial relationships

Using 2019 survey data, spatial regression modeling was used to investigate determinants of observed spatial patterns of stunting among children under the age of five. Spatial processes may operate at local or global scales. Accordingly, the OLS, GWR, and MGWR models were used to examine the relationships between under-five stunting and its predictors. To begin, the global OLS linear regression approach was used to forecast the variables' connection. The residuals of the OLS models were then examined for spatial autocorrelation using Moran's I statistics to ensure that they were not clustered. The robust probability revealed coefficient significance (*p* 0.01) for the explanatory factors, while the Joint Wald statistic suggested overall model significance (*p* 0.01). Furthermore, OLS regression results are only valid if the regression model meets all of the assumptions. The assumptions that needed to be met, the model performance, and the model significance were all tested while running the OLS regression. Furthermore, a certain independent variable may be a significant predictor in one cluster but not in another. This is non-stationary, and GWR can be used to detect it. Given a response and a set of predictor variables, GWR calibrates a regression model on each spatial unit of analysis and weights different regression parameters anywhere in the study area. GWR finds the best bandwidth for all covariates and simulates all processes at the same spatial scale. A positive coefficient means revealed that predictors and under-five stunting changed in the same direction and a negative coefficient indicates the existence of a vice versa relationship [38, 39]. Subsequently, GWR approach may be limited because it implicitly assumes the same spatial scale for each predictor, and these scales may be incorrect.

In contrast to GWR, a golden section search bandwidth was used to calibrate the MGWR model. For each predictor variable in an MGWR model, an individual bandwidth is determined. This permits each target-to-predictor variable relationship to have a different scale of relationship non-stationarity. Furthermore, the MGWR model calibration uses an iterative back-fitting procedure. Through optimal covariate-specific bandwidths, MGWR extends the GWR paradigm by allowing different processes to operate at different spatial scales [41–43]. We used the same set of variables included in the OLS model to specify GWR and MGWR models and to account for spatial non-stationarity in the relationships between childhood stunting and its predictors. To investigate models goodness of fitness, we compared model fitness using the Akaike information criterion (AIC), multiple *R*-squared values, and modified *R*-squared values which were used to compare the global and local models [43].

### Ethical considerations

The data were obtained by requesting it from the Demographic and Health Surveys (DHS) Program and can be freely accessed from the program website (www.dhsprogram.com). Before releasing data from the DHS to the public, it is de-identified, meaning that participants' identifiers are deleted and institutional ethical approval is waived, assuring compliance with human subject protection rules. In field surveys, GIS coordinates are only collected for the entire enumeration area (EA), not for individual houses, and the measured coordinates are randomly shifted throughout a vast geographic area, making it impossible to identify specific enumeration areas.

## Results

### Sample characteristics

The analysis included data from a weighted sample of 26,308 children under the age of 5 years, from their consecutive surveys conducted in 2011, 2016, and 2019. Overall, out of eleven regional states, the majority of children were from the Oromia region 11,348 (36.2%); on the other hand, Harari regional state had a minority of children 77 (0.3%). Regarding residence, about 2272 (83.8%) of them were from rural areas and the remaining 3793 (24.3%) children were urban residents. See Table 1 for a detailed description.

**Table 1:** Socio-demographic characteristics of the sample population (weighted, *N* = 26,308)

Characteristics	EDHS			
	2011 *n* (%)	2016 *n* (%)	2019 *n* (%)	Total *n* (%)
Sex				
Male	5584 (51.3)	5305 (51.1)	2673 (50.6)	13,562 (51)
Female	5299 (48.7)	5071 (48.9)	2605 (49.4)	12,975 (49)
Age in month				
< 6	1078 (9.9)	1108 (10.7)	523 (9.9)	2709 (10.2)
6–8	580 (5.3)	570 (5.5)	265 (5.0)	1415 (5.3)
9–11	499 (4.6)	500 (4.8)	228 (4.3)	1227 (4.6)
12–17	1002 (9.2)	1127 (10.9)	567 (10.7)	2696 (10.3)
18–23	910 (8.4)	892 (8.6)	471 (8.9)	2273 (8.6)
24–35	2043 (18.8)	1941 (18.7)	1052 (19.9)	5465 (19.1)
36–47	2450 (22.5)	2011 (19.4)	1168 (22.1)	5629 (21.3)
Above 48	2321 (21.3)	2224 (21.4)	1004 (19.0)	5549 (20.6)
Residence				
Rural	9541 (87.7)	9245 (89.1)	3941 (74.6)	22,727 (83.8)
Urban	1342 (12.4)	1131 (10.9)	1338 (25.4)	3793 (24.3)
Mothers educational status				
No education	7212 (66.3)	6533 (65.9)	2627 (53.6)	16,372 (61.9)
Primary	2797 (25.7)	2687 (27.1)	1731 (35.4)	7215 (29.4)
Secondary	229 (2.1)	471 (4.8)	360 (7.4)	1060 (4.8)
More than secondary	147 (1.4)	226 (2.3)	179 (3.7)	552 (2.5)
Wealth quintile				
Poorest	2452 (22.5)	2391 (23.0)	1188 (22.5)	5961 (22.6)
Poorer	2385 (21.9)	2415 (23.3)	1168 (22.1)	5968 (22.4)
Middle	2289 (21.0)	2161 (20.8)	1007 (19.1)	5457 (20.3)
Richer	2163 (19.9)	1927 (18.6)	940 (17.8)	5030 (18.8)
Richest	1593 (14.6)	1481 (14.3)	976 (18.5)	4050 (15.8)
Region				
Tigray	733 (6.7)	691 (6.6)	361 (6.8)	1785 (6.7)
Afar	105 (0.9)	98 (0.9)	77 (1.5)	277 (1.1)
Amhara	2324 (21.4)	2086 (20.1)	1001 (19)	5411 (20.2)
Oromiya	4723 (43.4)	4491 (43.3)	2134 (40.4)	11,348 (36.2)
Somali	278 (2.6)	417 (4.0)	359 (6.8)	1054 (4.5)
Benishangul-Gumuz	123 (1.1)	106 (1.0)	62 (1.2)	291 (3.3)
SNNP	2311 (21.3)	2188 (21.1)	1078 (20.4)	5577 (20.9)
Gambella	33 (0.3)	23 (0.22)	21 (0.4)	77 (0.3)
Harari	23 (0.2)	20 (0.2)	16 (0.3)	59 (0.23)
Addis Ababa	194 (1.8)	215 (2.1)	144 (2.7)	553 (2.2)
Dire Dawa	35 (0.3)	40 (0.4)	26 (0.5)	100 (0.4)
Total (*n*)	*n* = 10,883	*n* = 10,376	*n* = 5278	

### Temporal changes and burden of childhood stunting in Ethiopia from 2011 to 2019

Overall, across three surveys conducted between 2011, 2016, and 2019, the magnitude of stunting among children aged under 5 years decreased from 44.42% [95%, CI: 0.425–0.444] in 2011; 38.36% [95%, CI: 0.363–0.385] in 2016; and 36.77% [95%, CI: 0.349–0.375] in 2019 (Additional file 1).

### Spatial distribution of childhood stunting

About the spatial autocorrelation summary in Table 2, we employed the global autocorrelation statistics to assess geographical variation of childhood stunting in Ethiopia yearly from 2011 to 2019 (see Additional file 2). Accordingly, the outputs reveal the spatial distribution of childhood stunting across three waves of EDHS surveys was non-random. The observed regional clustering of stunting ranged from Moran's I index 0.81–0.35, and the Global Moran's I values were positive across surveys. Furthermore, in all surveys, the *Z* score was positive with a *p* value of 0.001, indicating that the observed clustering is less than 1% likely to be the result of random chance. The spatial autocorrelation analysis revealed the presence of statistically significant clusters at a 0.01, level of significance in each survey (Table 2).

**Table 2:** Spatial autocorrelation analysis of childhood stunting in Ethiopia

Survey	Moran’s *I*	*Z*-score	*p* value
EDHS, 2011	0.8195	25.92	< 0.000
EDHS, 2016	0.3968	12.98	< 0.000
EDHS, 2019	0.3530	7.734	< 0.000

#### Space-time burden of childhood stunting at regional level

**Fig. 1:**
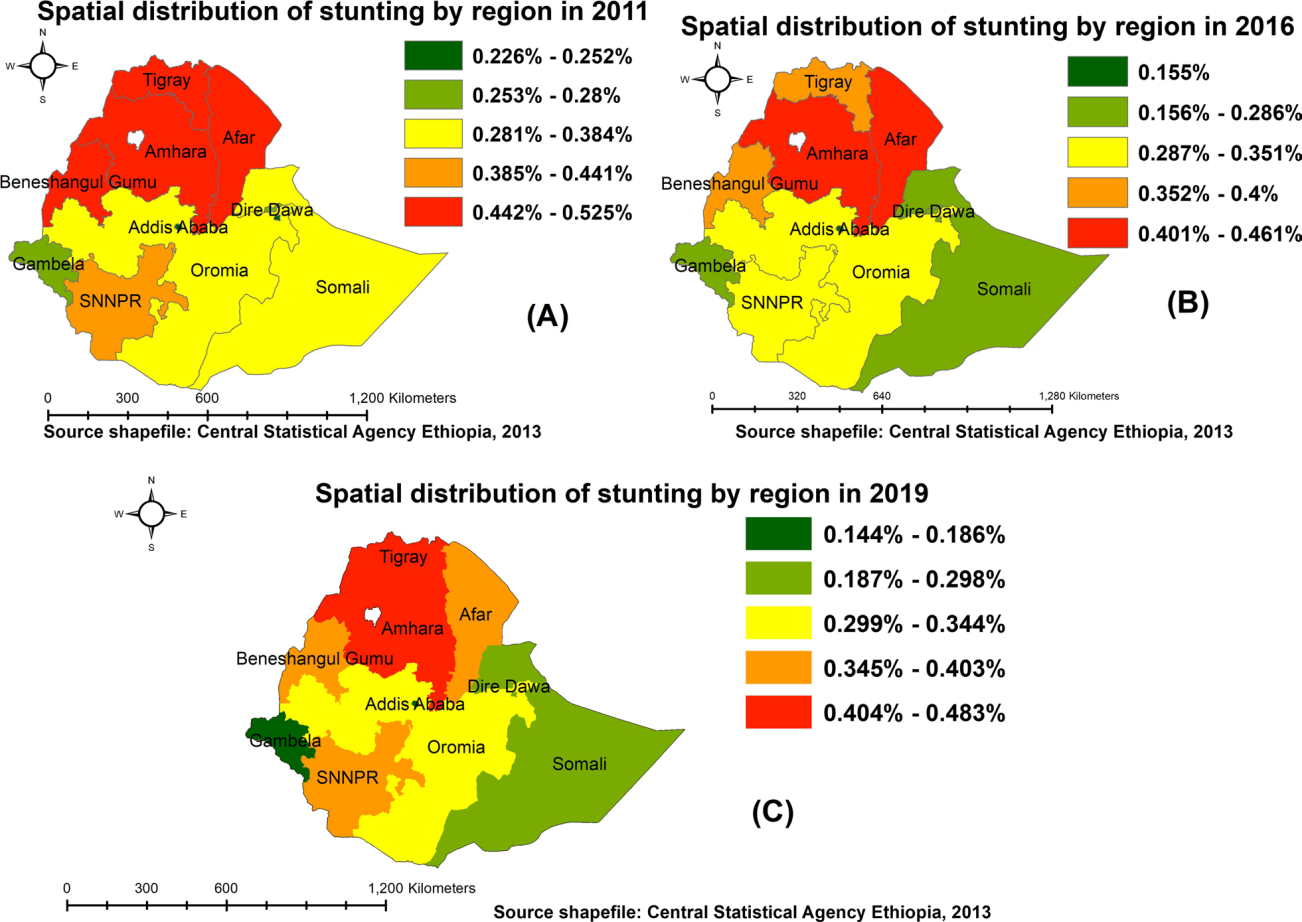
Spatial distribution of under-five stunting at regional level in Ethiopia: 2011 (**A**), 2016 (**B**), and 2019 (**C**)

In 2011 EDHS, the highest prevalence of stunting ranges from 44.2 to 52.5% was estimated in the northern parts of Ethiopia (Tigray, Amhara, Afar, and Benishangul-Gumuz), followed by parts of the SNNP region (35–44.1%). While the lowest prevalence of stunting was observed in parts of Addis Ababa and Dire Dawa (Fig. 1A). In the 2016 EDHS, the prevalence of stunting was significantly reduced in Tigray and Benishangul-Gumuz regions. However, the highest prevalence of childhood stunting was still observed in Amhara and Afar regions ranging from 40.1 to 46.1% (Fig. 1B). Despite the significant reduction of childhood stunting across all regions, the 2019 survey also indicated that northern parts of the country still share the highest burden of childhood stunting in Ethiopia (Fig. 1C). Overall across three waves of DHS, stunting in children under 5 years (Fig. 1) shows a marked belt of very high (close to 50% of children stunted) magnitude consistently in northern Ethiopia stretching in Tigray, Amhara, Afar, and Benishangul-Gumuz. Another area of very high stunting incidence was observed in Southern parts of Ethiopia.

#### Space-time burden of childhood stunting at the zone (district) level

**Fig. 2:**
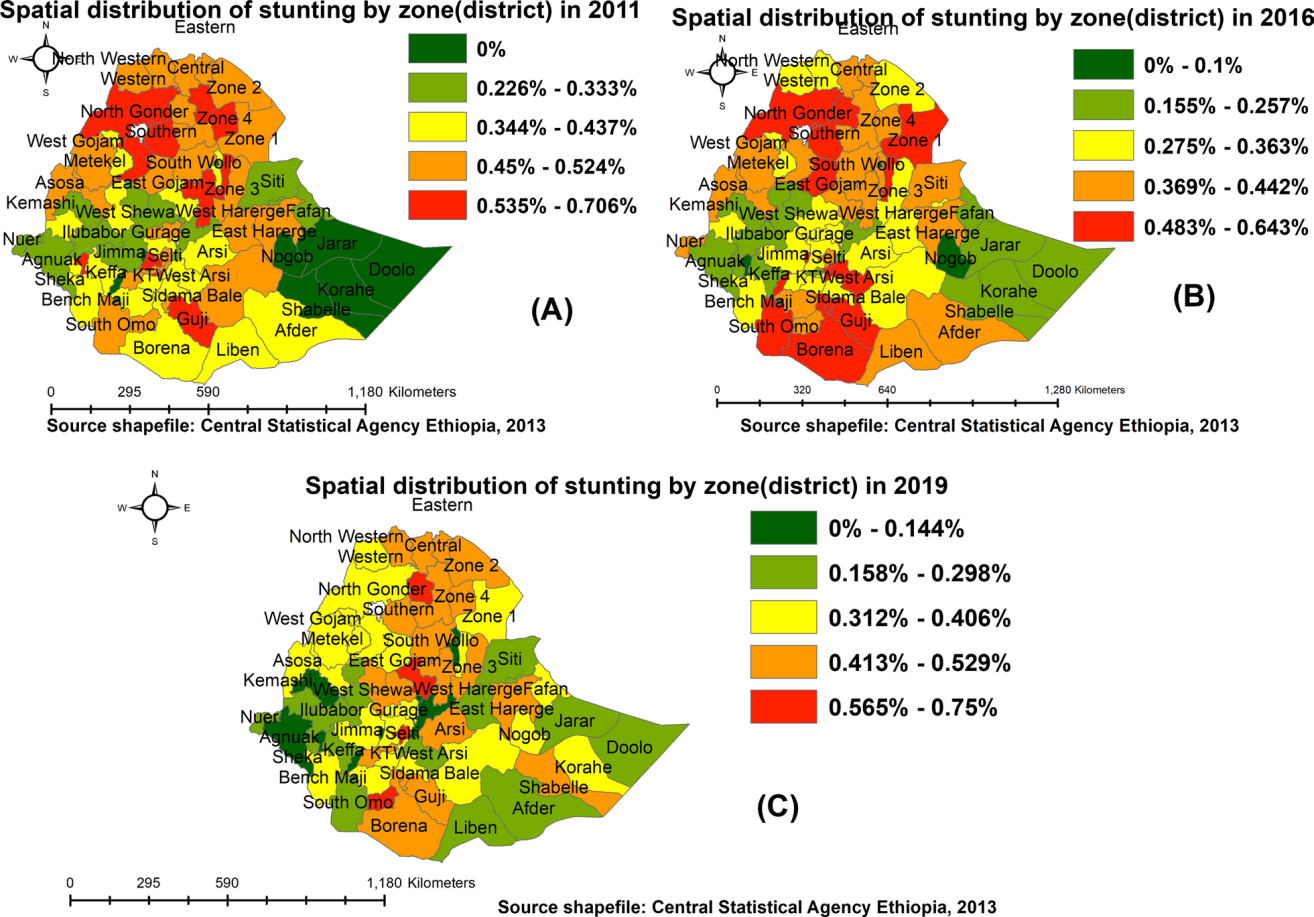
Spatial distribution of under-five stunting at districts level in Ethiopia: 2011 (**A**), 2016 (**B**), and 2019 (**C**)

District-level distribution of stunting in children under 5 years as mapped from three waves of EDHS data is shown in Fig. 2. There are pockets of extremely high stunting incidence in Ethiopia, and districts in the red map were among the highest proportion of stunted children across surveys (Fig. 2).

### Hot spot analysis

**Fig. 3:**
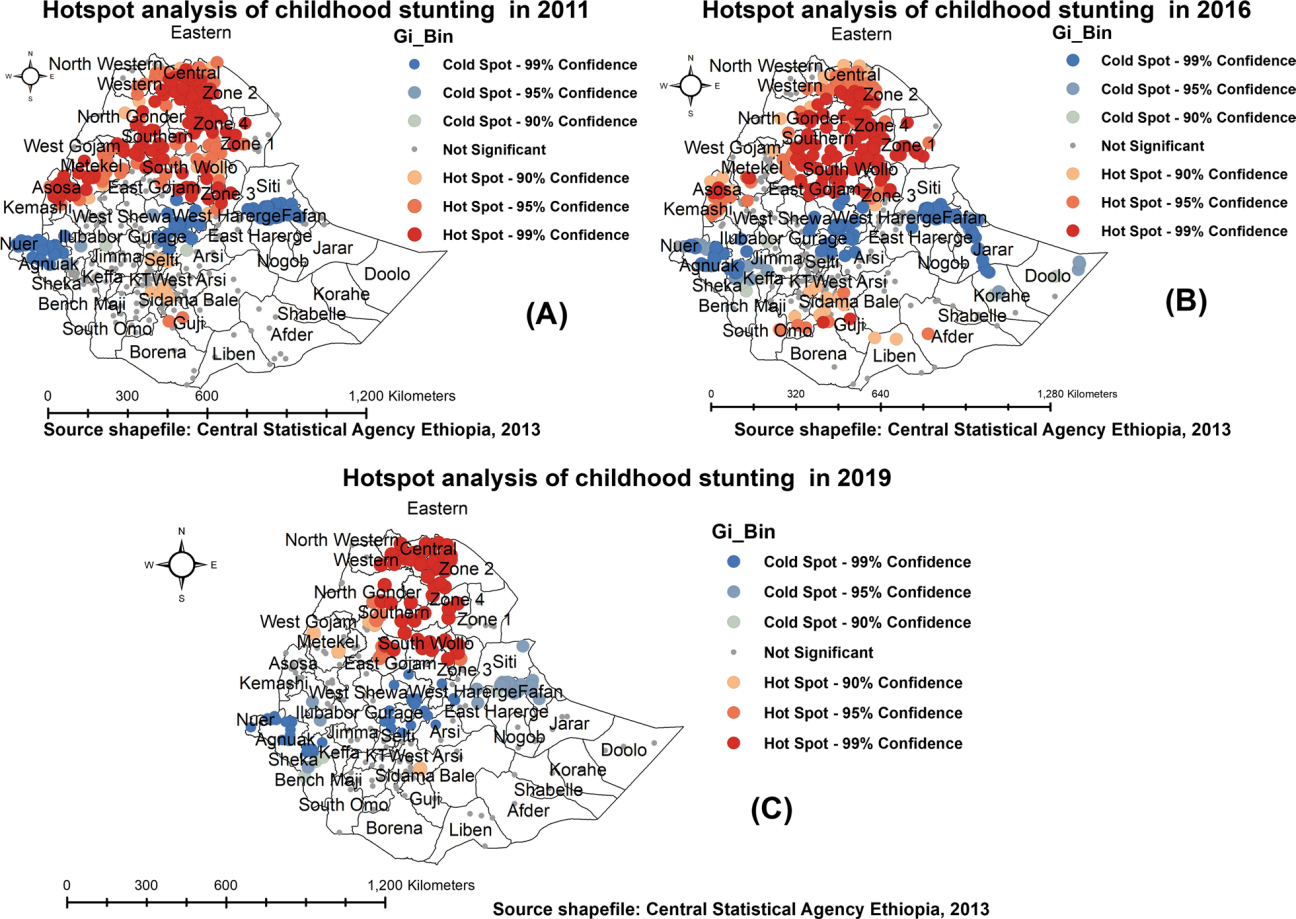
Hot spot analysis of under-five stunting in Ethiopia: 2011 (**A**), 2016 (**B**), and 2019 (**C**)

The Getis–Ord hot spot analysis identifies hot spots (areas with high cases) and cold spots where areas with low cases are surrounded by low cases. Figure 3 shows the hot spot and cold spot areas with the high and low stunting in children under 5 years in Ethiopia over the last fifteen years. The spatial clustering of hot spots (high risk) was consistently observed in the northern part of Ethiopia in all surveys. Furthermore, a high risk of childhood stunting was observed in southern and eastern parts of Ethiopia. For example, as can be seen in Fig. 3C in 2019 sampled data, a high prevalence of childhood stunting was detected in the Amhara region of East Gojjam, North and South Gondar zones, and South Wollo zone, and in the Sidama district of SNNP region, while parts of central Ethiopia, Dire Dawa, and Harari were identified as having a lower risk of stunting in children under 5 years.

### Spatial interpolation

**Fig. 4:**
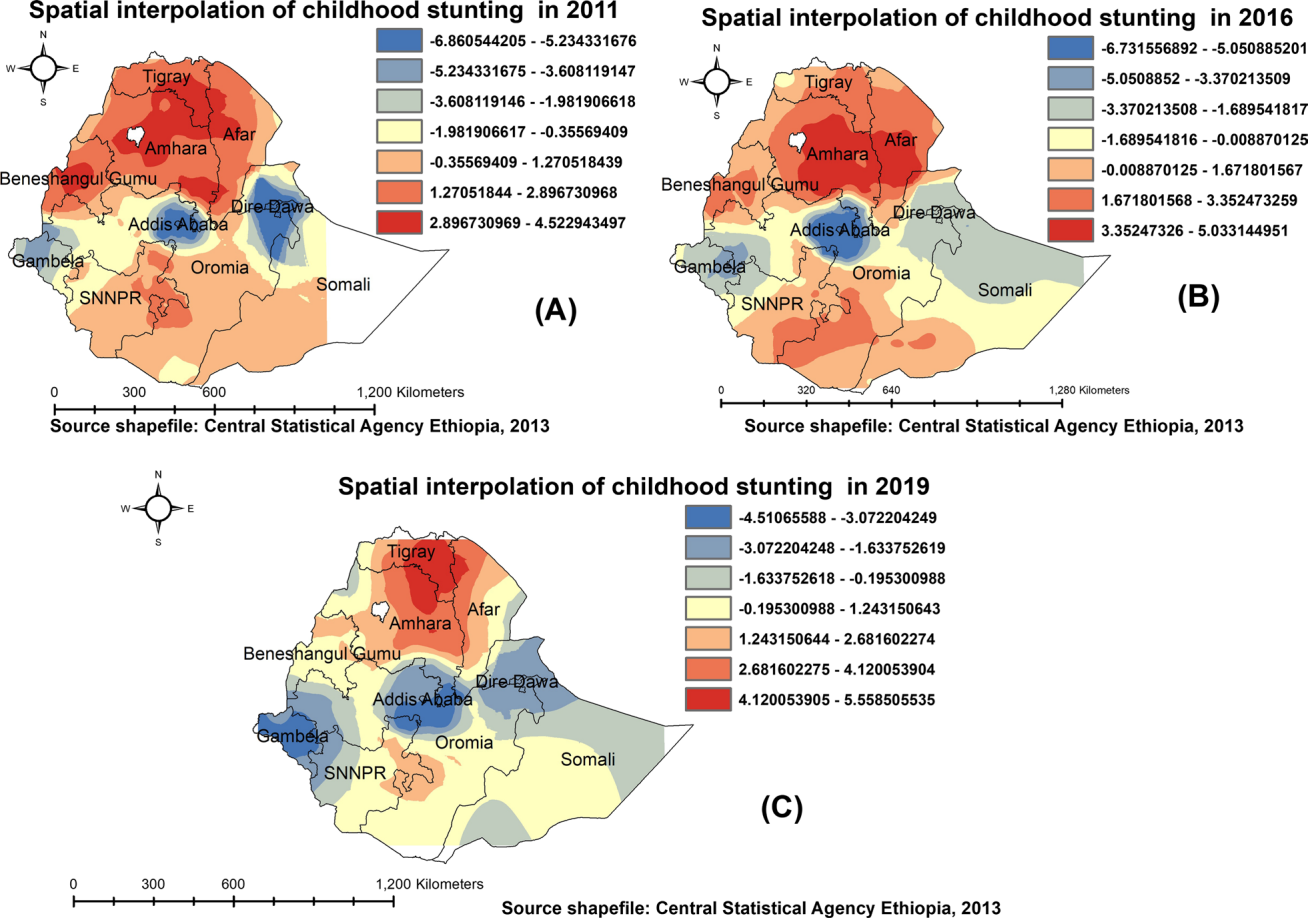
Spatial Interpolation of under-five stunting in Ethiopia: 2011 (**A**), 2016 (**B**), and 2019 (**C**)

Based on the observed data, we predicted geographical variations of childhood stunting in areas without observation by employing used the kriging interpolation. The produced risk maps highlight the areas with lower and high-risk childhood stunting. Overall across three waves of EDHS, our prediction map revealed that there is noticeable local geographical variation in terms of childhood stunting, with the risk of childhood stunting being elevated in the northern and southern, and few parts of the eastern district in Ethiopia. Highlighting the areas that are likely to have higher childhood stunting and mapping the risk of childhood stunting can help policymakers identify priority areas during the resource allocation process. In contrast, the predicted areas with a lower prevalence of childhood stunting were detected in central parts of Ethiopia (central Oromia and Addis Ababa), Dire Dawa, and west Gambella regions (Fig. 4).

### Sat scan analysis spatial scan statistical analysis

**Fig. 5:**
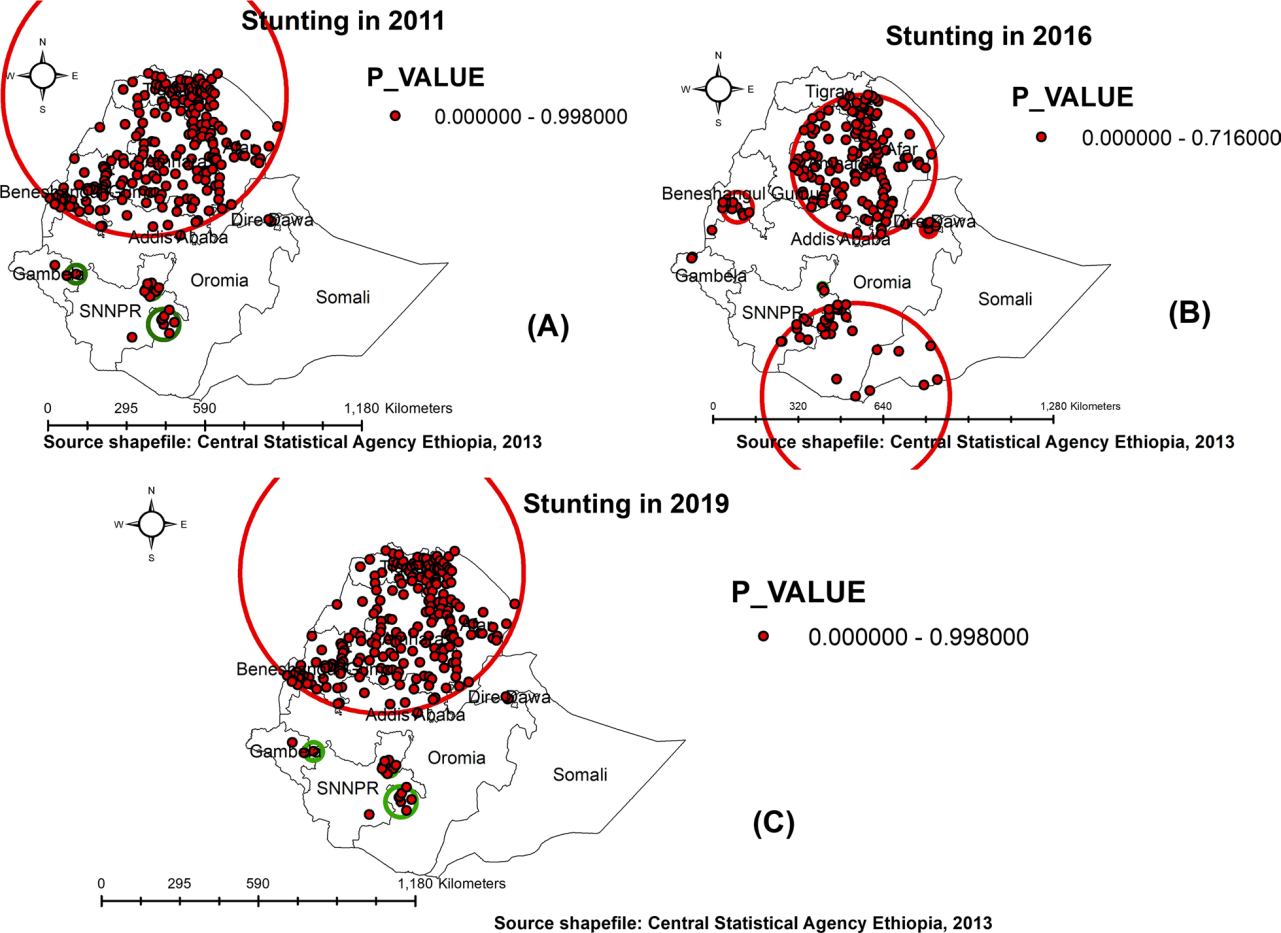
Sat scan cluster analysis of under-five stunting in Ethiopia: 2011 (**A**), 2016 (**B**), and 2019 (**C**)

Figure 8 depicts the results of a Sat scan analysis of Ethiopian childhood stunting throughout three waves of the EDHS. The primary cluster in the 2011 EDHS was found in the Amhara region (North and South Gondar zones, as well as the South Wollo zone), the South Tigray zone, and the Afar regional state. The primary cluster's geographical window was centered at 13.802279 N, 37.611555 E, with a radius of 531.73 km, a relative risk of 1.4, and a log-likelihood ratio of 97.42 at a *p* value of 0.001 (Fig. 5A). This suggests that children who live in this cluster are 50% more likely to be stunted than those who do not. The *p* value is high enough to rule out the possibility that this cluster was formed by chance.

The cluster's spatial window in the 2016 EDHS was centered at 11.451941 N, 39.572319 E, with a radius of 270.16 km, a relative risk (RR) of 1.38, and a log-likelihood ratio (LRR) of 55.1, at *p* 0.01, and a relative risk (RR) of 1.38. This suggests that children under the age of five who lived within the geographic window were 1.38 times less likely to be stunted than children under the age of five who lived outside the spatial window (Table 2). Secondary clusters were detected in the regions of south Oromia, southwest Somalia, Harari, and the east SNNPRs (Fig. 5B). According to the 2019 EDHS, the most likely cluster was found in northern Ethiopia, centered at 13.987653 N, 37.973902 E, with a radius of 553.46 km. The relative risk was 1.49 with a *p* value of 0.01, and the log-likelihood ratio was 187.827676 (Fig. 5C). It means that children under the age of five in the spatial circle were 1.49 times more likely to be stunted than children under the age of five outside the spatial circle.

### Spatial regression analysis

#### Ordinary least square

OLS model was employed to explore spatial regression assumptions and estimate variable coefficients of selected explanatory variables on under-five stunting. The OLS regression identified predictors of each hot spot of under-five stunting. Table 3 shows the results of the OLS model comparison. The Joint *F*-statistics and Joint Wald statistic outputs showed that our model was significant (*p* value 0.001). This model explained roughly 34.0% of the variation in stunting in children under the age of five (adjusted *R*2 = 0.34). Furthermore, the Koenker statistics outputs were [Koenker statistics = 4.06, *p* value 0.004], indicating that there was a non-stationary difference between stunting clusters and selected factors. Thus, we have employed the GWR model in our study as it considers that the relationship between independent and dependent variables is spatially heterogeneous across the area.

**Table 3:** OLS diagnostics summary result for under-five stunting using 2019 EDHS

Diagnostic criteria	Magnitude	*p* value
Number of observation	305	
AIC	− 269.87	
*R* squared	0.35	
Adjusted *R* squared	0.34	
Joint *F* statistics	32.18	0.000
Joint Wald statistic	238.66	0.000
Koenker (BP) statistic	4.06	0.004
Jarque–Bera statistic	6.00	0.496

Regarding the spatial regression summary in Table 4, our outputs indicated there was no multicollinearity among selected explanatory variables.

**Table 4:** Spatial regression summary result of OLS coefficients for under-five stunting in Ethiopia

Explanatory variables	Coefficient	Standard error	*t*-statistic	Probability	Robust probability	VIF
Intercept	0.244	0.037	6.56	0.000	0.000	
Proportion poor wealth index	− 0.542	0.1731	− 3.108	0.002	0.0124	1.184
Proportion poor sanitation	0.001	0.0002	3.120	0.001	0.000	1.820
Proportion inadequate diet	− 0.378	0.104	− 3.631	0.003	0.000	1.654
Proportion rural residents	0.07	0.023	3.029	0.002	0.005	1.483
Proportion uneducated mothers	0.237	0.033	7.136	0.000	0.000	1.377

#### Geographically weighted regression and multiscale extensions

Table 5 summarizes the diagnostic results of the GWR model. Different variable coefficients for variables discovered in OLS analysis were identified as a result of GWR. The GWR model explained almost 37% of the observed variability in stunting among under-five children in Ethiopia.

**Table 5:** Geographically weighted regression (GWR) model for under-five stunting in Ethiopia, EDHS 2019

*Variables*: the proportion of poor wealth index, poor sanitation, inadequate diet, rural residents, and undedicated mothers	GWR
Residual squares	6.28
Effective number	23.17
Sigma	0.149
AIC	− 280.232
Multiple *R*-squared	0.419
Adjusted *R*-squared	0.374

The summary statistics of estimated coefficients of the local terms (MGWR model), as well as the optimal bandwidth for each predictor and the result of the Monte Carlo test of non-stationarity, are described in Table 6. The result of the spatial heterogeneity test (Monte Carlo test of non-stationarity) shows a statistically significant result for the proportion of poor wealth index, poor sanitation, rural residents, and undedicated mothers (*p* < 0.05), implying spatial variability among these predictors.

**Table 6:** Summary of the MGWR model result among predictors of under-five stunting in Ethiopia

Explanatory variables	Mean	STD	Minimum	Maximum	Median	Non-stationarity (*p* values)
Proportion poor wealth index	− 0.169	0.063	− 0.266	− 0.060	− 0.175	0.010
Proportion poor sanitation	− 0.131	0.076	− 0.076	− 0.036	− 0.114	0.000
Proportion inadequate diet	0.066	0.131	− 0.098	0.272	0.005	0.104
Proportion rural residents	0.243	0.081	− 0.098	0.272	0.253	0.000
Proportion uneducated mothers	0.338	0.011	0.302	0.355	0.339	0.000

#### Performance comparison of the global and local spatial regression models

The OLS, GWR, and MGWR models were used to investigate the relationships between under-five stunting and its predictors. To begin, global spatial regression models were used to investigate geographical predictors of stunting in children under the age of five in Ethiopia. GWR and MGWR were then applied to the collection of variables found in the global models to investigate local spatial variation in the connections with the observed magnitude of stunting.

Table 7 summarizes the diagnostic comparison of the goodness-of-fit measures among global and local models. In terms of model definition and performance, we compared model fitness using the Akaike information criterion (AIC), multiple *R*-squared values, and modified *R*-squared values. As a result, the diagnostics of GWR revealed a relatively improved adjusted *R*2 and AIC, with the model *R*2 increasing to 0.37 and the AIC decreasing to − 280.23, both of which are much lower than the global model (− 269.87), indicating a superior fit. In addition, the MGWR model has the lowest AIC (− 735.99) and the highest *R*2 value (0.389). This means that the MGWR model approximately explained 39% of the variation in stunting among Ethiopian children under the age of five.

**Table 7:** Comparison of goodness-of-fit measures between global and local models

Outcome measure	OLS	GWR	MGWR
*Stunting among children under the age of 5 years*			
AIC	− 269.87	− 280.232	− 735.997
Multiple *R*2	0.35	0.419	0.423
Adjusted *R*2	0.34	0.374	0.389

Overall, the model diagnostics demonstrate that the MGWR model outperformed the OLS and GWR models significantly, implying that the MGWR model is statistically superior to the OLS and GWR models. A detailed explanation can be found in Table 7.

#### Factors affecting the spatial variation of childhood stunting

**Fig. 6:**
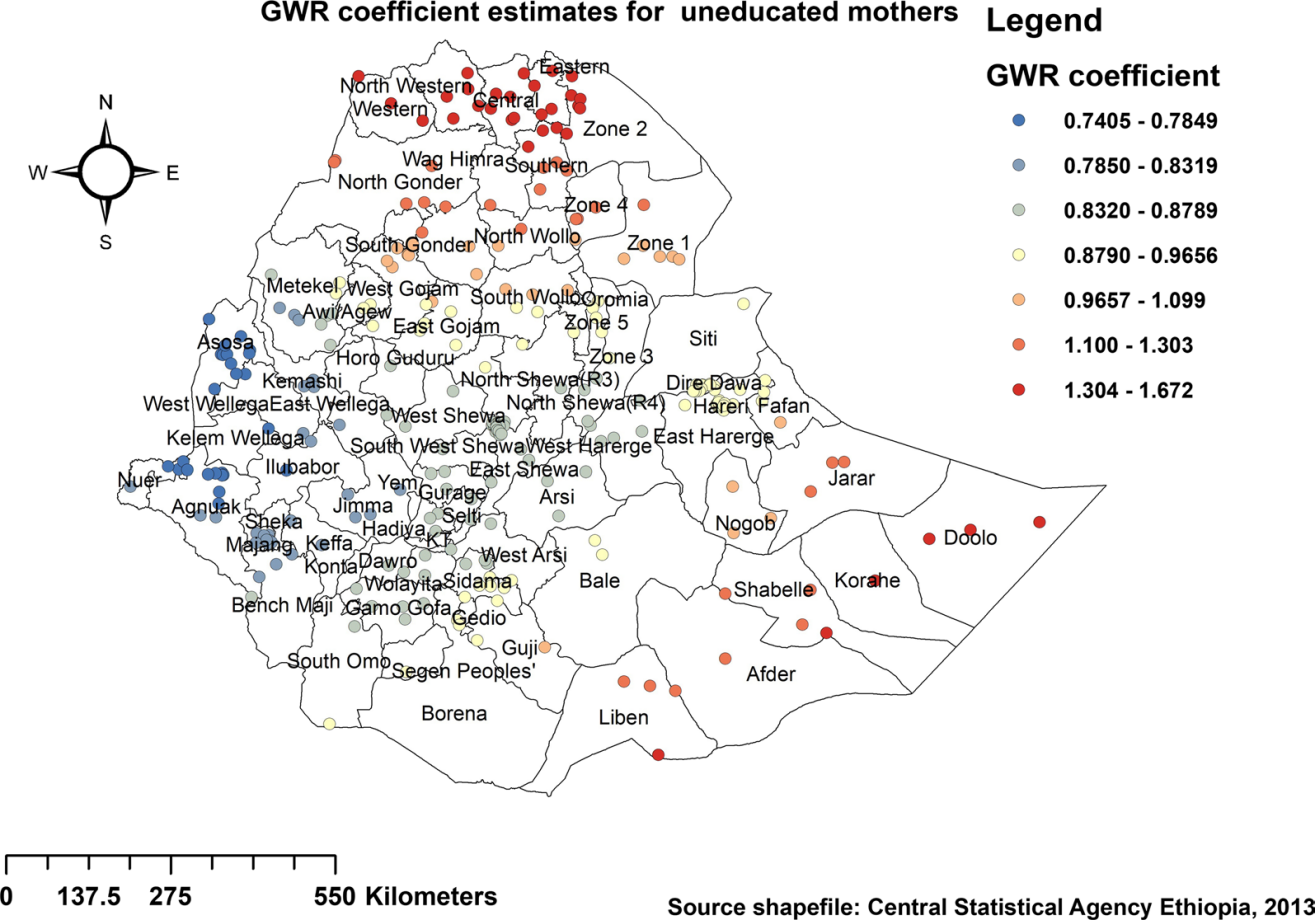
Spatial distribution and significance of mothers educational status on under-five stunting in Ethiopia

**Fig. 7:**
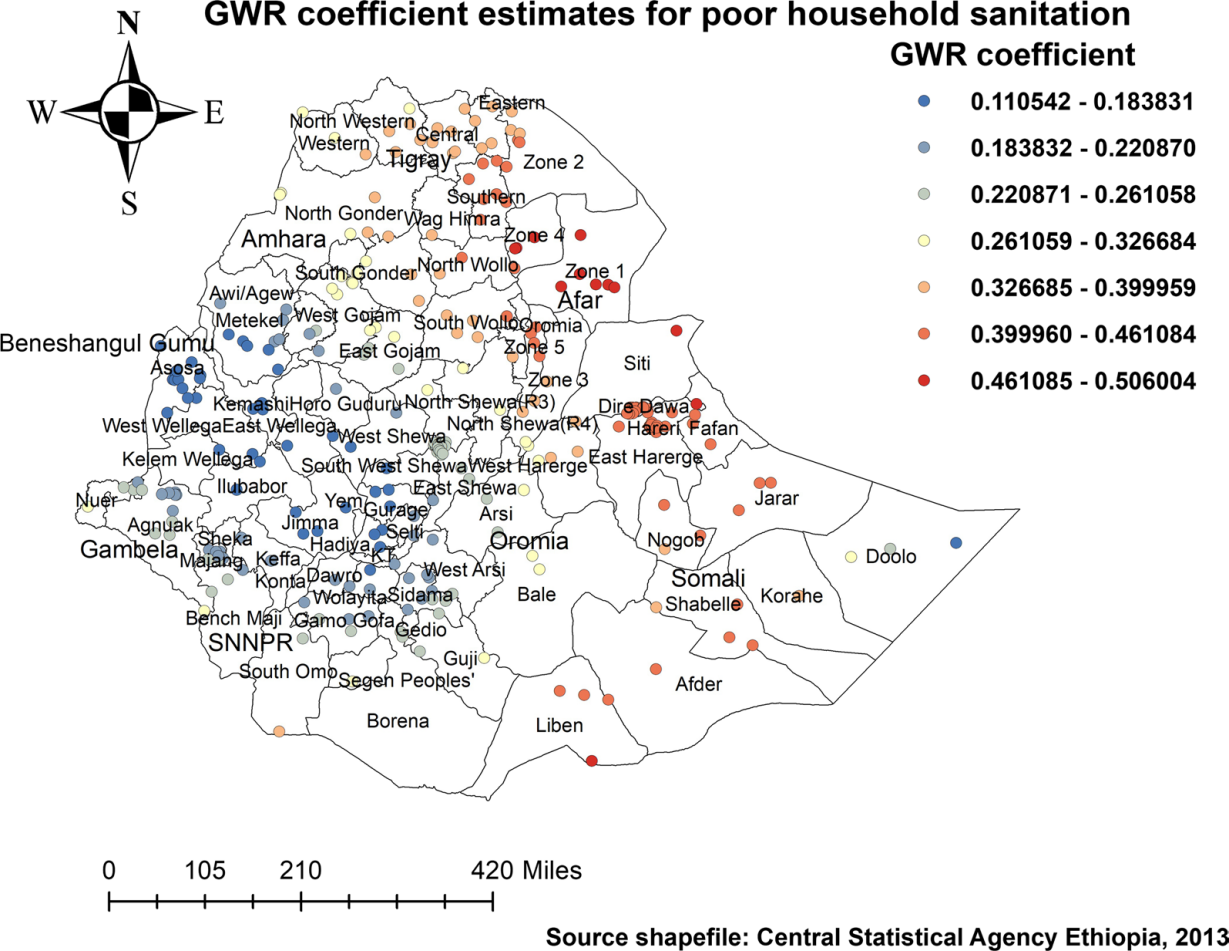
Spatial distribution and significance of poor sanitation on under-five stunting in Ethiopia

**Fig. 8:**
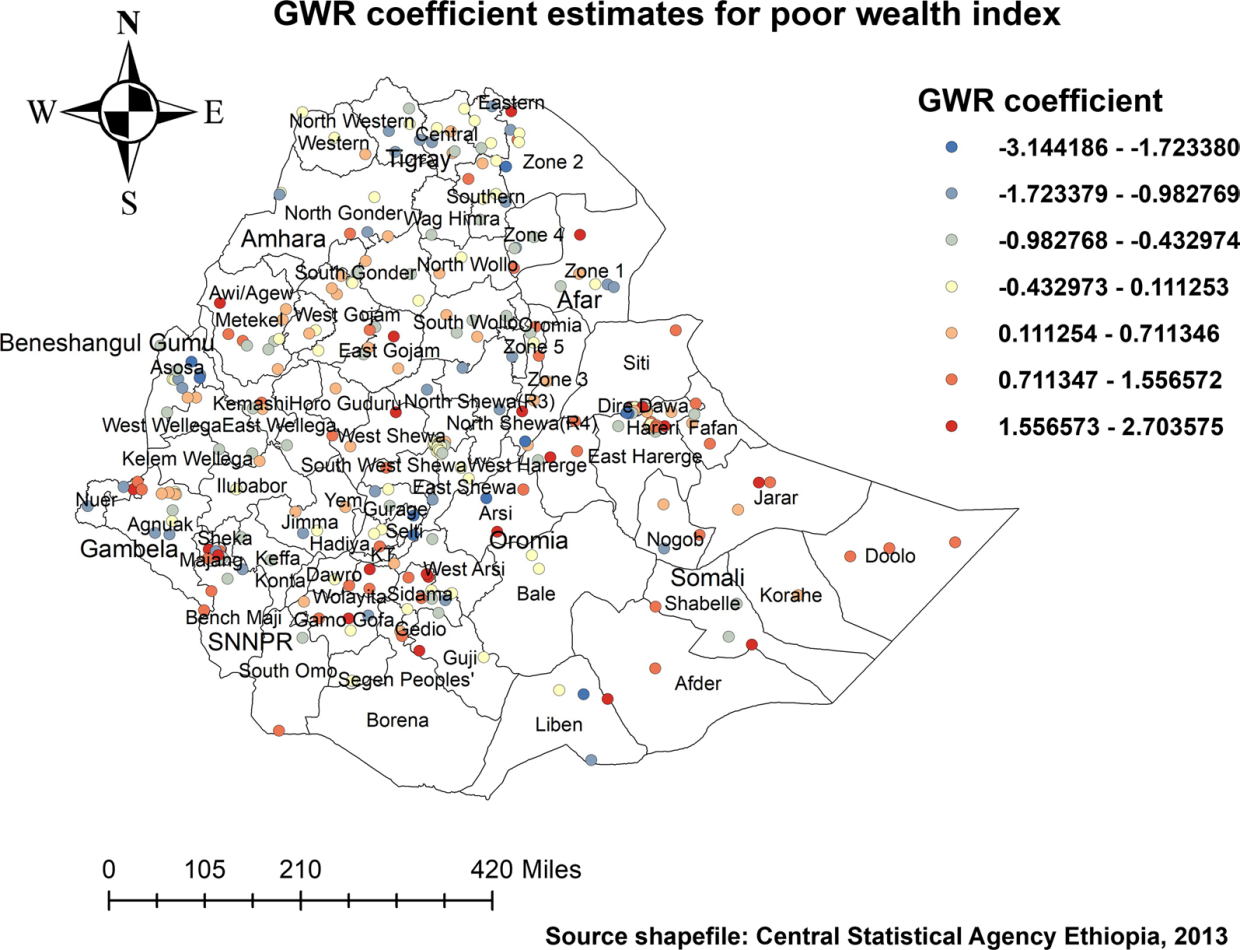
Spatial distribution and significance of wealth index under-five stunting in Ethiopia

**Fig. 9:**
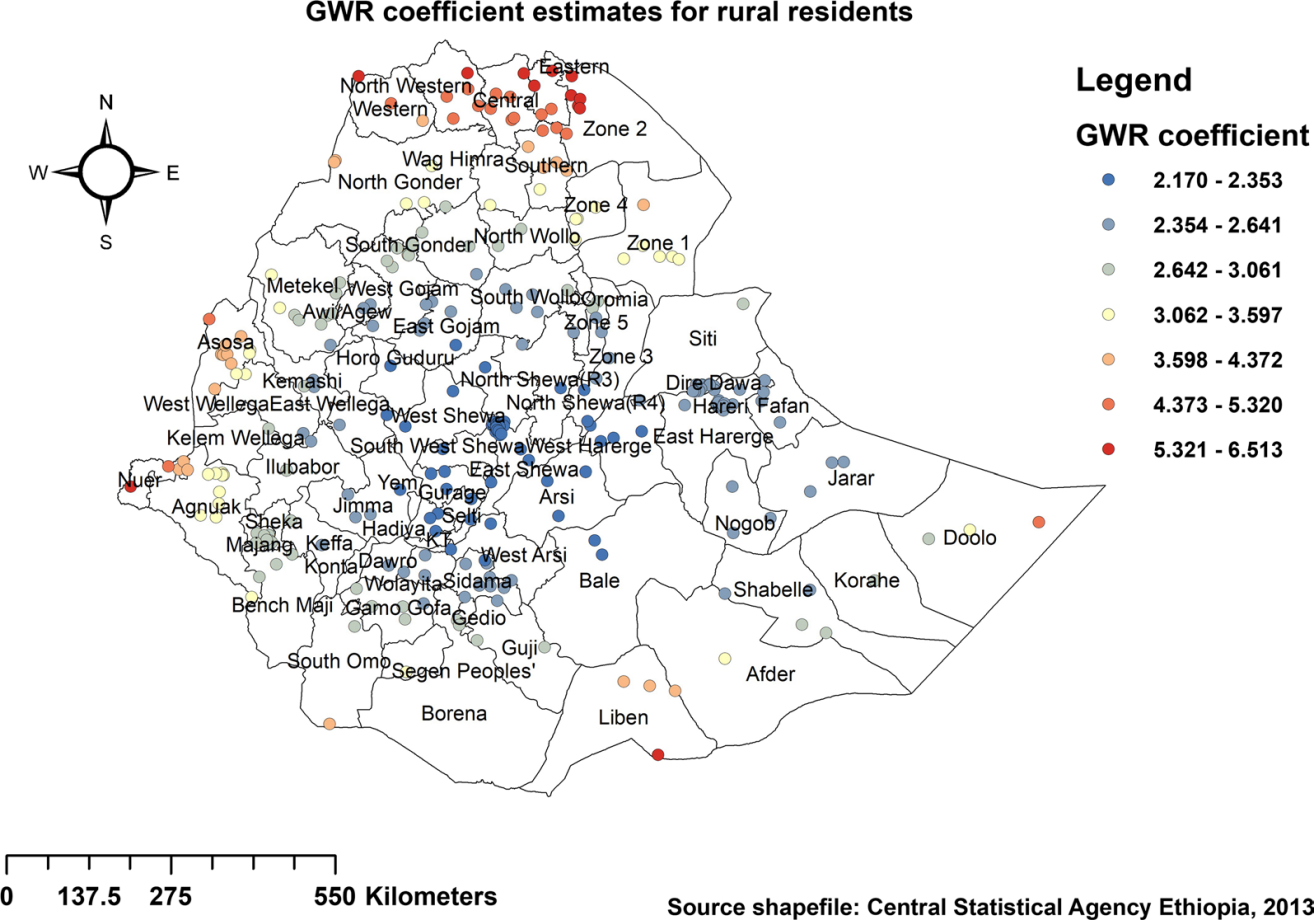
Spatial distribution and significance of residences on under-five stunting in Ethiopia

**Fig. 10:**
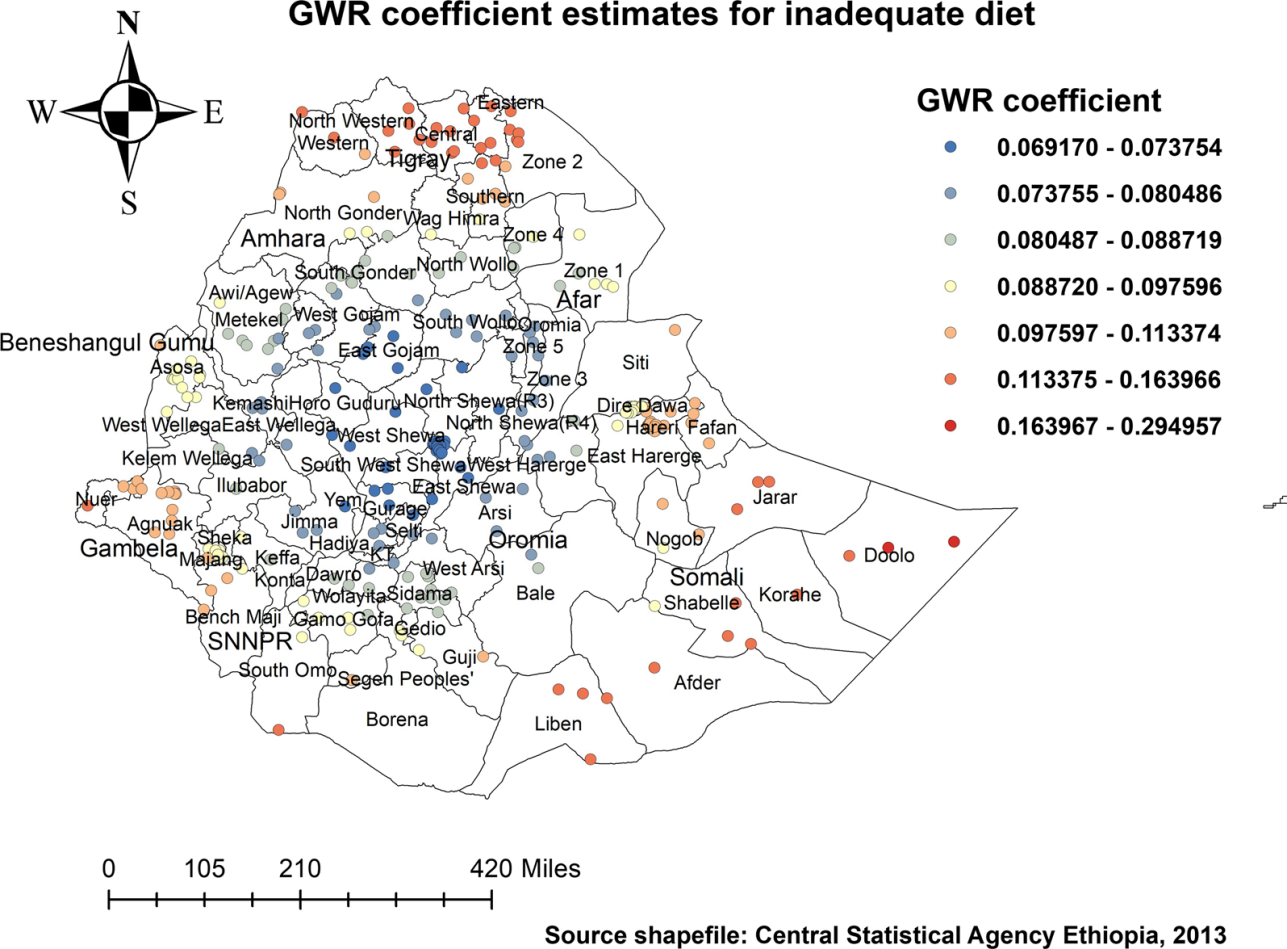
Spatial distribution and significance of inadequate diet on under-five stunting in Ethiopia

Regarding spatial predictors of stunting among under-five children in Ethiopia, GWR output produced predicted under-five stunting maps of geographic areas where mothers' education, household sanitation, household wealth index, residence, and diet were strong and weak predictors of under-five stunting as shown in Figs. 6, 7, 8, 9 and 10. The coefficients of uneducated mothers spatially vary from 0.74 to 1.67, which indicates a positive effect on stunting among under-five children in Ethiopia. As shown in Fig. 6, large portions of the Tigray and Somali regions were dominated by a positive significant association between uneducated mothers and stunting among children under the age of 5 years. However, higher coefficients for uneducated mothers were detected in parts of Amhara, Dire Dawa, SNNPR, Bale, Guji, and west and eastern parts of Hararghe in the Oromia region.

Furthermore, coefficients of children in households with poor sanitation spatially vary from 0.045 to 0.24; poor sanitation of the household shows a positive association with a higher proportion of stunting among children under the age of 5 years in eastern parts of Hararghe in the Oromia region, Afar, Dire Dawa, Harari, and the Somali region (Fig. 7).

Figure 8 presents a map of the coefficients for households with poor wealth index, indicating being under five in households with poor wealth index positive and strong predictor of stunting in some parts of the Amhara region, Benishangul-Gumuz, Gambella, parts of western and southern Oromia, and a few parts of SNNP region.

In addition, analysis of the results also highlighted the fact that under-five children residing in rural areas of Tigray, Afar, and parts of western Gambella regions were positively associated with under-five stunting (Fig. 9).

For a spatial variation of stunting among under-five children who do not receive an adequate diet, the results are significant in the eastern, southern, and central parts of Tigray, and parts of the Amhara region (mainly in the North Wollo, Waghmera, and Oromia zone) (Fig. 10).

**Fig. 11:**
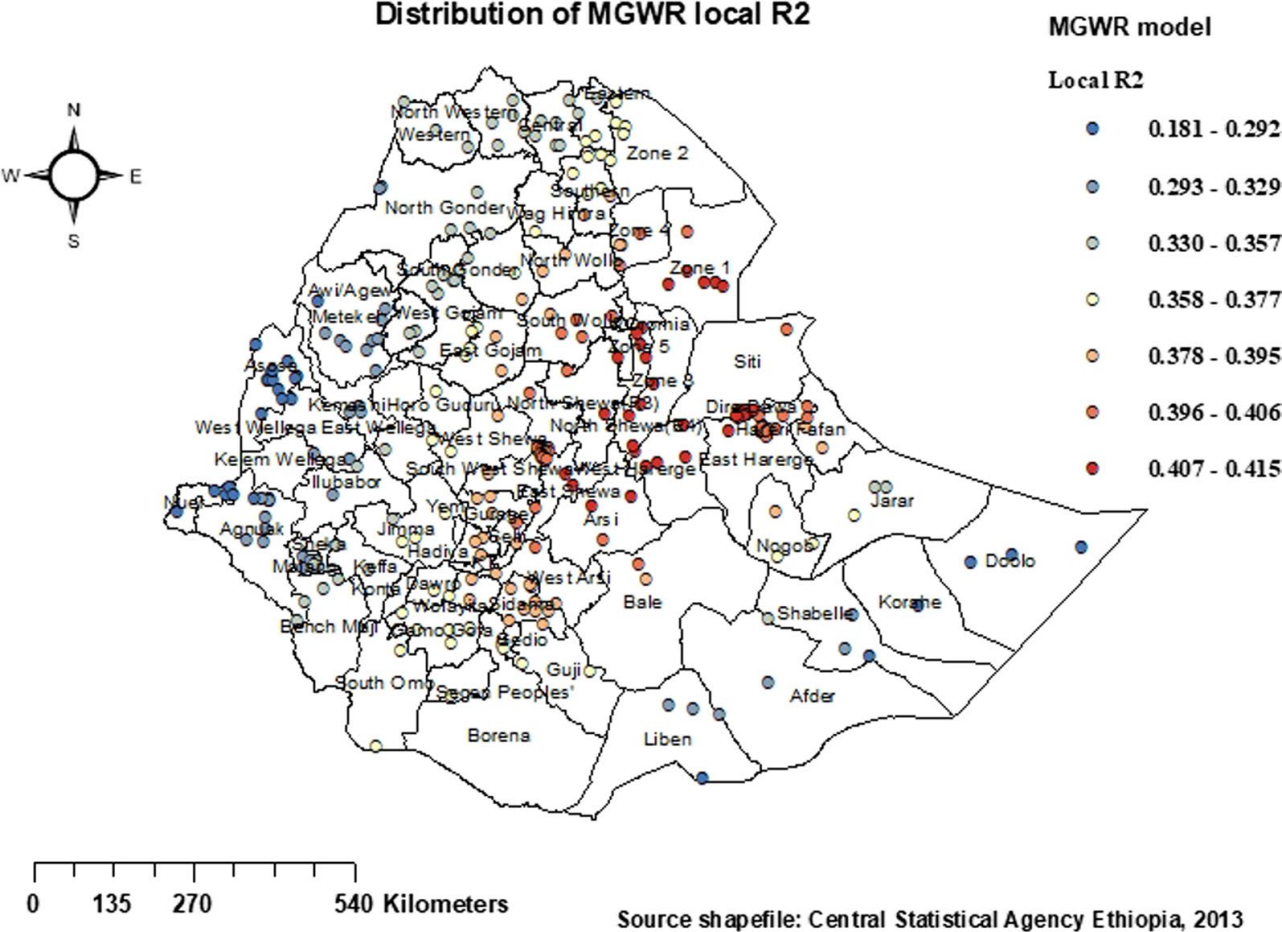
Distribution of MGWR local *R*^2^ for under-five children stunting in Ethiopia

Furthermore, the MGWR model was applied to the same set of predictors used in the GWR models in order to investigate the local geographic variation in the connections with the observed proportion of stunting among Ethiopian children under the age of five. Figure 11 illustrates the spatial distributions of local *R*2 values in the MGWR model, indicating a slightly lower performance of the model across these districts. As a result, the MGWR model has the lowest adjusted *R*2 (0.181), indicating that it explains at least 18.1% of the variation in stunting among Ethiopian children under the age of five. Several districts in the Amhara region, Afar, Tigray, Ethiopia's central area, Oromia's southern and eastern regions, and Dire Dawa and Harari have very high local *R*2. Local *R*2 values were low in a few districts in the Benishangul-Gumuz and Gambella regions, primarily in the Amhara region's Awi/Agew district and part of Ethiopia's Somali region, indicating that the model performed well in those districts.

## Discussion

The present study has examined the overall time trend and spatial trend and projection of stunting among children under the age of 5 years in Ethiopia and explored spatial predictors observed stunting variation by employing geospatial statistical models, based on EDHS data collected in 2011, 2016, and 2019. From 2011 to 2019, we observed the overall magnitude of under-five stunting significantly decreased from 44.42% [95%, CI: 0.425–0.444] in 2011 to 36.77% [95%, CI: 0.349–0.375] in 2019, and similarly reduction of under-five stunting was observed at the global level. But the observed level of magnitude in Ethiopia still lags far behind the country's plan of achieving the global SDGs of ending all forms of malnutrition [10, 11]. Conversely, this national prevalence of under-five stunting shows large disparities and variation across Ethiopia; for example, in 2019 the variation ranges from 14% in Addis Ababa to 49% in Tigray and varies more than six times across districts within the region. This is evidenced by our Moran's I statistic which suggested a presence of strong spatial heterogeneity of under-five stunting. Similarly, hot spot or Getis–Ord Gi* analysis revealed a significant clustering and correlation between under-five stunting and districts and regions of Ethiopia. This is supported by previous spatial analysis of under-five stunting conducted previously in Ethiopia [24, 25, 27–30]. Furthermore, in the other settings, various pieces of evidence revealed the presence of geographical variation of under-five stunting such as studies conducted in Rwanda [44], Ghana [18], Zambia [19], Nigeria [20, 21].

In this large population-based study, under-five children with stunting conditions were reported to be more clustered in most northern parts of Ethiopia and a similar pattern of stunting was also observed in some parts of southern and eastern Ethiopia. These areas were continuously presented as significant hot spots or clusters with a higher proportion of under-five stunting than children in central parts of Ethiopia such as Addis Ababa and parts in the Dire Dawa, and west Gambella. Our findings of hot spots or clusters areas rates at the district level of regions with a higher level of under-five stunting attest to the variations and support amenability of nutritional intervention. Therefore, policies are urgently needed to reduce the existing variation of under-five stunting across districts with greater burden, and making such priorities contributes to achieving a significant reduction in children stunting. Further, amenability to plausible decision-making and interventions would help to speed up the process of achieving the relevant SDG target [10, 11], in Ethiopia.

The results of spatial regression in the global model reveal that the regional distribution of stunting among Ethiopian children under the age of five is likely to be impacted by the proportion of uneducated mothers, poor household sanitation, poor household wealth index, and rural residents, and inadequate diet. Furthermore, to explore the local spatial variation in the relationships with the proportion of stunting among under-five children in Ethiopia, GWR and MGWR were applied to the same set of predictors used in the global models. Both the GWR and MGWR approach improved our understanding of the association between childhood stunting and its predictors, but the MGWR model had the smallest AIC value (− 735.99) and the highest *R*2 value (0.389). Therefore, MGWR is statistically preferable and it revealed varied importance of factors at the local level compared to other models. This is in line with previous findings [23].

Findings indicated poor household sanitation and poor household wealth index exhibited a predominantly positive influence on under-five children to be stunted. This is also evidenced in some literature [19, 22, 44]. Similarly, when analyzing potential factors that may affect the clustering of under-five stunting, stunting among children in Ethiopia was positively associated with children who had uneducated mothers. Within the literature, previous evidence also recognizes that there is a higher chance of educated women in terms of reducing child malnutrition [19, 22, 31]; it is often that these women who are more educated had improved access to media and health-seeking practices, which further promotes the better dietary practice. Our GWR analysis revealed a positive relationship (both strong and weak positive relationships) between residences of participants, with being a rural resident being a strong predictor of disparity in under-five stunting. In the eastern Tigray region, the southern and eastern parts of Amhara, and the Afar regions of Ethiopia, being a rural resident was a strong predictor of under-five stunting, as shown in Fig. 9. Furthermore, the MGWR model shows that the proportion of poor wealth index, poor sanitation, undedicated mothers, and rural resident were significantly associated with stunting in Ethiopia. Similarly, a study conducted in Uganda stated mothers education and household wealth index as a significant predictor of local geographical variation of stunting [23].

The current study has various limitations that must be noted when interpreting the results. First, the study's cross-sectional design hinders inferring causation between the explanatory and outcome variables. Also, the survey replies may be disposed to participant recall bias. Despite its limitations, this study has an enormous number of strengths. Firstly, nationally representative data were used along with appropriate geospatial statistical methods that took into account complex survey design and sample weights. The spatial predictors of stunting among children under the age of 5 years were identified by using the 2019 latest national survey data which is the first of its kind in Ethiopia. Furthermore, trends of prevalence across districts and regions were assessed using these nationally representative data. Thus, we believe that our findings and suggestions will have tremendous contributions to spatial predictors of childhood stunting to a gap in the literature regarding its spatial predictors, and further, this study contributes to a better understanding of the under-five stunting burden and disparities in Ethiopia.

## Conclusion

Using a robust geospatial technique, this paper presents the time and geospatial trend of stunting among children under the age of 5 years in Ethiopia. Also, it has been able to explore spatial predictors of observed stunting variation by employing both global and local geospatial regression models. Hence, this study provides comprehensive evidence of under-five stunting trends, geographic-based inequalities, and spatial predictors in Ethiopia.

In Ethiopia, overall progress has been made in decreasing stunting among children under the age of 5 years; although disparities varied in some areas and districts between surveys, the pattern generally remained constant over time. These findings suggest a need for region and district-specific policies where priority should be given to children under the age of 5 years where most likely to exhibit high-risk stunting. The observed decreases are less pronounced in districts identified as hot spots continuously across surveys, and these require further policies targeting those districts. Further, future interventions and investigations may consider the role of identified factors in local GWR analysis which is predominantly related to the socioeconomic and cultural background of participants in our study to reduce the observed disparities and burden of stunting.

## Supplementary Information

**Additional file 1: Table S2. **Significant spatial clusters of stunting among under-five children in Ethiopia, EDHS 2011, 2016, 2019.

**Additional file 2: **The global spatial autocorrelation figures.

## Data Availability

The data we used for this analysis are publically available through the MEASURE DHS initiative, which you can find at www.measuredhs.com. The dataset can be accessed and downloaded freely after describing the study's goals.

## Abbreviations

CSA: Central Statistical Agency
DHS: Demographic and Health Survey
EA: Enumeration area
EDHS: Ethiopia Demographic and Health Survey
SSA: Sub-Saharan Africa
GWR: Geographically weighted regression
MGWR: Multiscale geographically weighted regression
SDG: Sustainable Development Goals
SNNPR: Southern Nations, Nationalities, and Peoples' Region
OLS: Ordinary least square method
