# Supplementary material for: Spatial trends and projections of chronic malnutrition among children under 5 years of age in Ethiopia from 2011 to 2019: a geographically weighted regression analysis
Source: J Health Popul Nutr. 2022 Jul 5;41:28. doi: 10.1186/s41043-022-00309-7 (PMC9254552; doi:10.1186/s41043-022-00309-7)
Supplement: Supplementary file 1 — Additional file 1: Table S2. Significant spatial clusters of stunting among under-five children in Ethiopia, EDHS 2011, 2016, 2019. [file 41043_2022_309_MOESM1_ESM.docx]

| **Table 2 Significant spatial clusters of stunting among under-five children in Ethiopia, EDHS 2011, 2016, 2019** | | | | | | | | |
| --- | --- | --- | --- | --- | --- | --- | --- | --- |
| **Years** | **Cluster** | **Enumeration areas(clusters detected)** | **Coordinates /radius** | **Population** | **Cases** | **RR** | **LLR** | **P-value** |
| 2011 | 1 | 64, 592, 77, 637, 213, 249, 620, 462, 460, 174, 139, 650, 365, 347, 274, 634, 148, 180, 217, 333, 86, 195, 504, 403, 556, 488, 122, 154, 46, 638, 406, 311, 245, 316, 50, 247, 510, 131, 521, 467, 334, 241, 271, 227, 417, 582, 498, 287, 322, 235, 461, 280, 226, 515, 621, 183, 192, 482, 568, 177, 601, 636, 538, 597, 218, 246, 260, 71, 123, 497, 224, 480, 542, 615, 69, 493, 288, 21, 115, 35, 188, 419, 181, 543, 551, 533, 469, 225, 463, 319, 318, 418, 473, 20, 369, 453, 39, 66,299, 581, 133, 89, 300, 433, 635, 426, 231, 281, 171, 189, 79, 230,484, 407, 191, 557, 330, 585, 67, 439, 306, 445, 99, 372, 554, 388, 91, 65, 550, 84, 296, 90, 617, 94, 119, 18, 478, 43, 253, 499, 555, 293, 193, 414, 356, 29, 54, 68, 623, 367, 106, 366, 151, 302, 58, 437, 136, 398, 269, 579, 397, 118, 166, 438, 209, 392, 255, 628, 104,420, 219, 600, 643, 33, 164, 1, 38, 112, 232, 229, 476, 577, 204,589, 411, 622, 157, 423, 215, 6, 276, 142, 62, 83, 349, 325, 514, 314, 341, 625, 449, 512, 100, 329, 604, 278, 87, 421, 578, 607, 619, 85, 26, 40, 332, 443, 317, 563 | (13.802279 N, 37.611555 E) / 531.73 km | 4040 | 2082 | 1.38 | 97.42 | < 0.000 |
| 2016 | 1 | 410, 496, 611, 345, 18, 354, 616, 254, 189, 191, 571, 478, 591, 617, 368, 401, 389, 200, 455, 241, 55, 332, 344, 545, 547, 348, 249, 460,97, 351, 627, 276, 66, 488, 620, 176, 442, 544, 300, 38, 570, 310, 10, 267, 136, 283, 637, 334, 449, 128, 599, 143, 205, 102, 392, 542, 206, 199, 499, 178, 79, 295, 37, 120, 510, 135, 132, 572, 456, 482, 336, 511, 160, 512, 130, 229, 484, 24, 201, 440, 39, 172, 624, 632,424, 423, 350, 596, 628, 403, 427, 421, 538, 163, 158, 384, 167, 605,550, 564, 237, 94, 327, 152, 429, 531, 75, 220, 575, 73, 430, 230, 382, 312, 169, 375, 425, 218, 585, 51, 474, 99, 80, 298, 623, 579, 121, 431, 4, 235, 640, 516, 322, 49, 127, 491, 355, 196 | (11.451941 N, 39.572319 E) / 270.16 km | 2019 | 939 | 1.38 | 55.10 | < 0.000 |
|  | 2 | 65, 335, 569, 124, 621, 563, 209, 88, 433, 407 | (10.038797 N, 35.233469 E) / 57.12 km | 206 | 113 | 1.52 | 14.69 | 0.00032 |
|  | 3 | 453, 557, 441, 594, 166, 30, 473 | (9.303716 N, 41.792392 E) / 29.03 km | 146 | 84 | 1.59 | 13.54 | 0.00092 |
|  | 4 | 82, 7, 601, 377, 394, 422, 398, 208, 21, 316, 50, 182, 574, 232, 34,342, 32, 520, 600, 445, 405, 468, 503, 86, 556, 313, 634, 87, 466, 518, 450, 576, 505 | (3.621391 N, 39.291912 E) / 352.78 km | 684 | 311 | 1.27 | 12.56 | 0.0022 |
| 2019 | 1 | 8, 1, 9, 6, 7, 22, 13, 12, 21, 11, 2, 14, 56, 10, 16, 4, 23, 17, 5, 3, 15, 82, 83, 25, 84, 78, 20, 35, 36, 39, 24, 55, 85, 18, 27, 37,38, 57, 19, 62, 58, 59, 61, 74, 54, 81, 75, 46, 29, 53, 60, 45, 44,65, 70, 76, 165, 71, 63, 79, 51, 34, 162, 80, 64, 72, 66, 52, 33, 77,163, 30, 73, 148, 159, 47, 48, 67, 166, 31, 158, 49, 161, 68, 160,100, 164, 119, 26, 50, 32, 99, 43, 69, 167, 149, 168, 40, 150, 169,156, 42, 98, 146, 256 | (13.987653 N, 37.973902 E) / 553.46 km | 1938 | 878 | 1.49 | 59.37 | <0.000 |
|  | 2 | 170 | (9.532762 N, 34.455099 E) / 0 km | 24 | 19 | 2.23 | 9.51 | 0.017 |
|  | 3 | 266, 295, 48, 381, 250, 240, 346, 627, 5, 416, 93, 233, 187, 56 | 4.240002 N,41.906017 E/ 321.91 km | 92 | 91 | 1.13 | 8.6 | <0.032 |
| **EDHS, Ethiopian Demographic and Health Survey; E, East; KM, Kilometre; LLR, Log-Likelihood Ratio; N, North; RR, Relative Risk. N.B : A cluster is statistically significant when its LLR is greater than the critical value.** | | | | | | | | |
